# Unlocking the Dietary Puzzle: How Macronutrient Intake Shapes the Relationship between Visfatin and Atherosclerosis in Type 2 Diabetes

**DOI:** 10.3390/medicina60030438

**Published:** 2024-03-06

**Authors:** Kati Kärberg, Alastair Forbes, Margus Lember

**Affiliations:** 1 Institute of Clinical Medicine, University of Tartu, L. Puusepa 8, 50406 Tartu, Estonia; alastair.forbes@ut.ee (A.F.); margus.lember@ut.ee (M.L.); 2 Internal Medicine Clinic, Tartu University Hospital, L. Puusepa 8, 50406 Tartu, Estonia

**Keywords:** atherosclerosis, visfatin, intima–media thickness, type 2 diabetes mellitus, dietary patterns, nutrients

## Abstract

*Background and Objectives*. Optimal nutrition for type 2 diabetes (T2DM) aims to improve glycemic control by promoting weight loss and reducing adipose tissue, consequently improving cardiovascular health. Dietary alterations can influence adipose tissue metabolism and potentially impact adipocytokines like visfatin, thereby affecting atherosclerosis development. This study aimed to investigate dietary habits and adherence to recommendations among individuals with T2DM and to examine how dietary adherence influences the association between visfatin and subclinical atherosclerosis. *Materials and Methods*: This cross-sectional multicenter study involved 216 adults (30–70 years) with T2DM, assessing dietary habits, adherence to recommendations (carbohydrates, fats, protein, fiber, saturated fatty acid, polyunsaturated and monounsaturated fatty acid (PUFA and MUFA) and salt), and the association between visfatin and subclinical atherosclerosis. Participants completed 24 h dietary recalls; dietary misreporting was assessed using the Goldberg cut-off method. Carotid intima–media thickness (IMT) and plaque occurrence were evaluated with ultrasound, while visfatin levels were measured using Luminex’s xMAP technology. *Results*: Three of the eight recommendations were followed in 31% of subjects, two in 26%, and four in 20%, with the highest adherence to MUFA and protein intake. Significant correlations between IMT and visfatin were observed in individuals with specific dietary patterns. The association between IMT and visfatin persisted when PUFA and MUFA intake aligned with recommendations. PUFA intake ≤ 10% and MUFA ≤ 20% of total energy significantly correlated with carotid artery IMT (*p* = 0.010 and *p* = 0.006, respectively). Visfatin’s associations with IMT remained significant (*p* = 0.006) after adjusting for common risk factors, medication use, and dietary nonadherence. No association was observed with carotid artery plaque. *Conclusions*: Dietary compliance was limited, as only 31% adhered even to three of eight recommendations. A common dietary pattern characterized by low carbohydrate and fiber but high fat, total fat, saturated fat, and salt intake was identified. This pattern amplifies the statistical association between visfatin and subclinical atherosclerosis.

## 1. Introduction

Diabetes is evolving into a significant challenge within the field of medicine [1]. Medical nutrition therapy is pivotal in diabetes management, particularly in preventing diabetic complications, focusing on optimal body weight and cardiovascular health [2,3,4]. The 2023 nutritional guidelines do not reach a unanimous agreement on the optimal nutritional strategy or ideal dietary prescription, emphasizing the significance of personalized approaches.

The American Diabetes Association rejects a “one-size-fits-all” eating plan, favoring an individualized approach with intensive lifestyle intervention and personalized macronutrient distribution [4,5]. The European Association for the Study of Diabetes accepts varied carbohydrate intakes, as long as guidelines for dietary fiber (at least 35 g per day), sugars (<10% of total energy), saturated fats (<10% of total energy), and protein (10–20% of total energy) are followed [3].

The Mediterranean diet is considered the most suitable dietary approach for individuals diagnosed with type 2 diabetes mellitus (T2DM), featuring the following recommended dietary guidelines: 40–50% carbohydrates; 15–25% proteins; 25–35% fats (with less than 7% saturated, 10% polyunsaturated, and 10% monounsaturated fats); a minimum of 14 g of fiber per 1000 kcal consumed; and sodium intake limited to less than 2300 mg [2,3,4,5].

An adequately structured diet positively impacts patient well-being by supporting the upkeep of metabolic control and preventing and treating chronic complications associated with diabetes and mortality [6,7].

The appropriate amount of carbohydrates for maintaining health in patients with T2DM is still uncertain, as studies vary significantly in their approaches and recommended percentage ranges [2,5,8]. However, general nutritional recommendations with relative consensus say that individuals with T2DM should target 40–50% of their total energy intake from carbohydrates to satisfy the brain’s glucose requirements [5,8,9].

One of the most debated aspects of nutritional research concerns fats, particularly essential fatty acids, in the context of the risk of cardiovascular disease (CVD) [6,10,11,12,13]. Dietary fat is essential for energy production, the transportation of lipid-soluble vitamins, and overall physiological functions [14]. A higher total fat intake was associated with a lower risk of CVD but equivalent all-cause mortality [6]. The consumption of monounsaturated fatty acids (MUFAs) and polyunsaturated fatty acids (PUFAs) has been associated with a lower risk of CVD and mortality, while intake of saturated fatty acids (SFAs) and trans fats was linked to an increased risk of CVD [6]. Importantly, replacing SFAs with PUFAs lowered the risk of coronary heart disease by 10% for every 5% increase in energy intake [15]. Studies have also demonstrated a 37% lower risk of CVD when substituting SFA with MUFA intake [6].

Alterations in dietary intake, including inadequate and excessive consumption, significantly impact intricate interactions among the liver, neuronal, and hormonal systems [16]. The regulation of liver energy metabolism is also modulated by adipose tissue through the secretion of various proinflammatory and anti-inflammatory adipokines, such as leptin, adiponectin, resistin, and visfatin, along with cytokines and chemokines such as TNF-a, IL-6, etc. [17]. Visfatin, initially described in 2005 by Fukuhara, has multiple biological actions beyond the adipose tissue [18,19]. It has been posited as an indicator of cell proliferation, monocyte/macrophage activation and recruitment, vascular inflammation and remodeling, and endothelial dysfunction, representing an essential initial stage in the development of the atherosclerotic process in T2DM [19,20,21]. Therefore, visfatin may be a marker for atherosclerosis.

Previous studies have demonstrated that the macronutrient distribution in the diet may influence arterial stiffness in individuals with type 1 diabetes [22]. Research investigating the influence of dietary factors on serum visfatin levels in T2DM is notably limited, and the existing evidence is contradictory and requires additional investigation [23,24,25,26,27,28].

This study aims to evaluate the dietary habits of individuals with type 2 diabetes, adherence to dietary recommendations, and the effect of dietary adherence on the association between visfatin and subclinical atherosclerosis.

## 2. Materials and Methods

### 2.1. Population

Within the framework of a cross-sectional multicentric investigation, 216 individuals diagnosed with T2DM were enlisted. Recruitment spanned from November 2014 to March 2017, with participants sourced from 13 general practices in Estonia. T2DM diagnosis followed the World Health Organization’s 2006/2011 criteria: fasting plasma glucose ≥ 7.0 mmol/L (126 mg/dL), 2 h plasma glucose ≥ 11.1 mmol/L (200 mg/dL), or HbA1c ≥ 6.5% (48 mmol/mol) [29,30].

The research encompassed participants aged 30–70 who could autonomously attend outpatient clinics, had no prior diagnosis of atherosclerosis or its complications, and maintained a weight below 140 kg (the upper limit of the dual-energy X-ray absorptiometer). Exclusion criteria comprised other types of diabetes, pregnancy or lactation, a history of coronary artery, carotid artery, peripheral artery, or cerebrovascular disease, and a background of chronic inflammatory disease, malignant tumor, or other severe illness.

This research adhered to the principles of the Declaration of Helsinki and its subsequent amendments. Approval for this study was granted by the Research Ethics Committee of the University of Tartu (protocol number: 223/T-17; 25 February 2013). Written informed consent was obtained from each study participant, and stringent measures were implemented to anonymize all patient details.

### 2.2. Clinical and Biochemical Measurements

Participants were scheduled for morning appointments after a minimum of 10 h of fasting, during which they provided written informed consent before individual interviews. Information on medical history, smoking habits, and current medication was gathered using a non-validated questionnaire. A general practitioner verified participants’ medical histories and current prescription medication usage through the national electronic health information and prescription system. Individuals with antiplatelet and anticoagulant therapies were excluded from the analysis due to their dual usage for ischemic complications. Furthermore, therapy with acetylsalicylic acid is not regulated in Estonia and is available for purchase without a prescription. The analysis solely considered medications dispensed through pharmacies.

Participant height, waist circumference, and weight were measured with precision to the nearest 0.1 cm, 0.1 cm, and 0.1 kg, respectively. Subsequently, their body mass index (BMI) was computed by dividing their weight in kilograms by the square of their height in meters (kg/m^2^).

Seated blood pressure readings were collected from each patient using a standard mercury sphygmomanometer. Statistical analysis relied on the average of three measurements.

Venous blood samples were collected to assess levels of HbA1c, total cholesterol, triglycerides, low-density lipoprotein cholesterol (LDL-C), high-density lipoprotein cholesterol (HDL-C), and high-sensitivity C-reactive protein, utilizing routine laboratory methods. Serum levels of visfatin were determined using Luminex’s xMAP technology.

Hypertension was defined as systolic blood pressure ≥140 mmHg and/or diastolic blood pressure ≥90 mmHg, a history of hypertension, or current antihypertensive drug treatment, and hyperlipidemia was defined as LDL-C ≥ 2.6 mmol/L (≥100 mg/dL) or statin use [31].

The International Physical Activity Questionnaire (short form) was employed to evaluate the individual physical activity levels of the subjects. Activity was quantified as a continuous variable, represented by the metabolic equivalent of weekly task minutes [32]. This study adhered to the STROBE guidelines in its reporting [33].

### 2.3. Dietary Assessment

During the initial encounter, all study participants underwent a 24 h dietary recall session conducted by the investigator. The NutriData food composition database and dietary analysis software were utilized to incorporate the average nutritional composition of foods commonly consumed in Estonia [34]. Considering various cooking methods, the software translated this information into 60 nutrient equivalents. The specifics of the methodology for identifying misreporting are outlined also in a previously published article [35]. As overall nutrient intake is closely correlated with the amount of energy consumed, we used implausibly low energy intake to indicate a general under-reporting of food eaten. These subjects were excluded from the analysis to reduce bias. The Goldberg cut-off method was applied [36].

Reported energy intake (EIrep) was derived from the 24 h dietary recall. The Katch-McArdle Formula [BMR = 370 + 21.6 × weight × (100 − body fat)/100] was employed to compute the estimated basal metabolic rate (BMRest). This formula utilizes body weight in kilograms and body fat percentage. Body fat percentage measurements were obtained using a Lunar Prodigy Advance Dual Energy X-ray absorptiometer (G.E. Healthcare, Waukesha, WI, USA).

To evaluate the physical activity level (PAL), the ratio of reported energy intake (EIrep) to estimated basal metabolic rate (BMRest) was computed (PAL = EIrep:BMRest). This approach aligns with the principle that energy intake should match energy expenditure [36]. The International Physical Activity Questionnaires (IPAQ) were employed to gauge the subjects’ physical activity levels. Activity was quantified as a continuous variable (metabolic equivalent of weekly task minutes) and categorized as low, moderate, or high activity levels [32].

The IPAQ-derived categories were translated into PAL categories, where low, moderate, and high activities were assigned PAL values of 1.4, 1.6, and 1.8, respectively. Each physical activity category’s age-specific thresholds (95% lower and upper confidence limits) were calculated. For individuals aged 16 to 70, PAL ranges of 0.872 to 2.249, 0.996 to 2.570, and 1.120 to 2.892 were established for low, moderate, and high activity levels, respectively [37].

Individual PAL values were computed and juxtaposed with the corresponding activity category thresholds. Participants with an EIrep:BMRest ratio falling below the lower limit of their PAL were categorized as under-reporters. Plausible reporters were identified as individuals with an EIrep:BMRest ratio between the lower and upper limits. Over-reporters were defined as subjects with an EIrep:BMRest ratio exceeding the upper limit.

### 2.4. Nutritional Recommendations

The macronutrient target values were established following Estonia’s general dietary recommendations and the guidelines provided by The Diabetes and Nutrition Study Group of the European Association for the Study of Diabetes [9,38].

### 2.5. Measurement of Carotid Artery Intima–Media Thickness

Subclinical atherosclerosis in the carotid artery was characterized using carotid artery intima–media thickness (IMT). Measurements were performed by a single sonographer using a high-resolution B-mode tomographic ultrasound system (Philips Affiniti 70 G, Philips Healthcare, 3000 Minuteman Road, Andover, MA, USA) with a 12 MHz linear transducer.

To provide a comprehensive view of vessel orientation, plaque, and surrounding structures, a transverse scan was conducted from the proximal part of the common carotid artery (CCA) extending through the bulb to the distal internal carotid artery (ICA) and external carotid artery. Longitudinal scanning techniques generated two parallel echogenic lines, representing the intima and media layers. In plaque-free areas, measurements were taken from anterior, lateral, and posterior imaging planes in the CCA (10 mm proximal), bulb, and ICA (5–10 mm from the bulb).

Within each section of the carotid artery, three manual measurements were taken at a lower resolution (without utilizing the zoom function) from the far wall on both sides of the artery, resulting in 54 measurements per patient. Images and cine-loops were acquired using a three-lead electrocardiogram and saved as dynamic sequences in Digital Imaging and Communications in Medicine format for offline analysis with a RadiAnt DICOM Viewer. The same expert reviewed and measured the recordings, with an intra-observer variability for measuring IMT assessed at 3.5% through Bland–Altman analysis.

Consistent with the guidelines set forth by the American Society of Echocardiography and the Mannheim Carotid Intima–Media Thickness and Plaque consensus statement, subclinical atherosclerosis was characterized by an IMT exceeding 1 mm. Plaque was defined as focal wall thickening at least 50% greater than the surrounding area or as focal regions with an IMT greater than 1.5 mm [39,40]. The presence of plaque was recorded as a binary yes or no. In this context, atherosclerosis was defined as an IMT exceeding 1 mm or the presence of plaque.

### 2.6. Measurement of the Android-to-Gynoid Fat Ratio

The android and gynoid fat percentage assessments were conducted using a Lunar Prodigy Advance dual-energy X-ray absorptiometry machine (G.E. Healthcare, Waukesha, WI, USA). All scans adhered to the manufacturer’s recommended positioning, and a qualified and experienced technician executed measurements. Software provided by the manufacturer defined the regions of interest for regional body composition. The android-to-gynoid fat ratio (A/G) was calculated as the quotient of android fat divided by gynoid fat. A risk factor was identified when the A/G ratio exceeded 1.

### 2.7. Statistical Analysis

The sample size was determined based on a margin of error of 0.05, a population size of 1.329 million, and an anticipated population proportion of 8%. The calculations for sample size were performed using the following formulas:

n = [z^2^ × Φ(1 − Φ)]/ε^2^

n’ = n/{1 + [z^2^ × Φ(1 − Φ)]/ε^2^N}

where z is the z-score (2.58 for a confidence level of 99%), ε is the margin of error, n is the sample size within an unlimited population, n’ is the finite sample size, N is the population size, and F is the population proportion. The sample size was sufficient for all measured variables to achieve a statistical power greater than 0.99.

Continuous variables are expressed as means ± standard deviation (SD), while categorical variables are presented as percentages. Differences between categorical variables among groups were assessed using the chi-squared test or Fisher’s exact test (if the assumptions of the chi-square test were not met). ANOVA, or Student’s *t*-test, was applied to compare means for parametrically distributed variables. A linear regression model was constructed to examine the association between IMT, adherence to dietary recommendations, and other risk factors. All statistical tests were two-tailed with a 5% significance level, and adjustments were made for multiple comparisons using Bonferroni correction. Statistical analyses were performed using SPSS (IBM Corp. Released 2020. IBM SPSS Statistics for Windows, Version 29.0., Armonk, NY, USA).

## 3. Results

### 3.1. Characteristics of the Study Population

Table 1 presents the clinical characteristics of the participants. Following the study invitation, 254 patients attended family doctor appointments. Of these, 18 eligible patients opted not to participate, and 20 did not meet the inclusion criteria. Consequently, 216 patients were deemed eligible for this study. Four participants were excluded from the analysis during statistical assessments as outliers, verified through Cook’s distance and leverage values.

The study participants were categorized into two groups according to reported food intake: under-reporters and plausible reporters. These groups were approximately evenly distributed. Notably, there was only one over-reporter who was included in the plausible group. This individual constituted a mere 0.4% of the study population and did not exhibit outlier characteristics. The subsequent analyses are grounded in data derived from plausible respondents. 

### 3.2. Adherence to Dietary Recommendations

**Figure 1:**
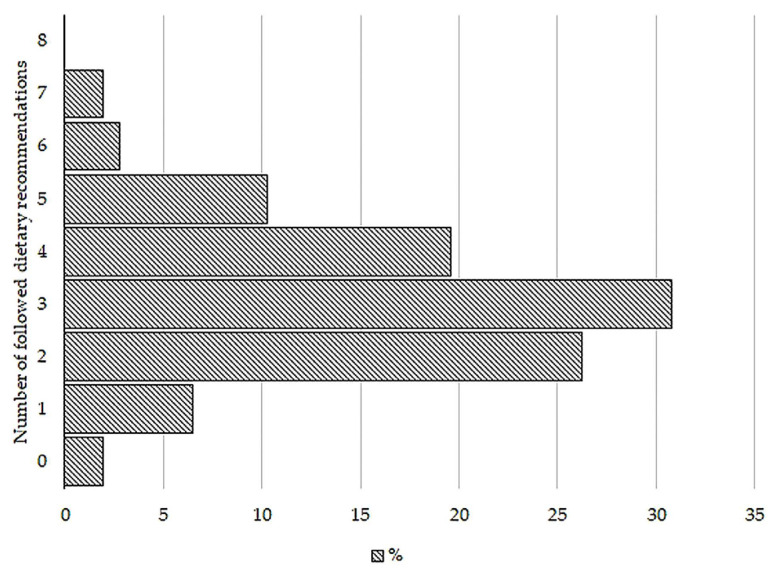
The quantity of followed recommendations. Abbreviations: %; the percentage of subjects who adhered to recommendations. Recommendations that were evaluated are as follows: carbohydrate 50–60%, fat 25–35%, protein 10–20%, saturated fat acid ≤ 10%, monounsaturated fatty acids 10–20%, polyunsaturated fatty acids 5–10% of total energy, fiber ≥ 14 g/1000 kcal, and sodium ≤ 2.3 g/day.

**Figure 2:**
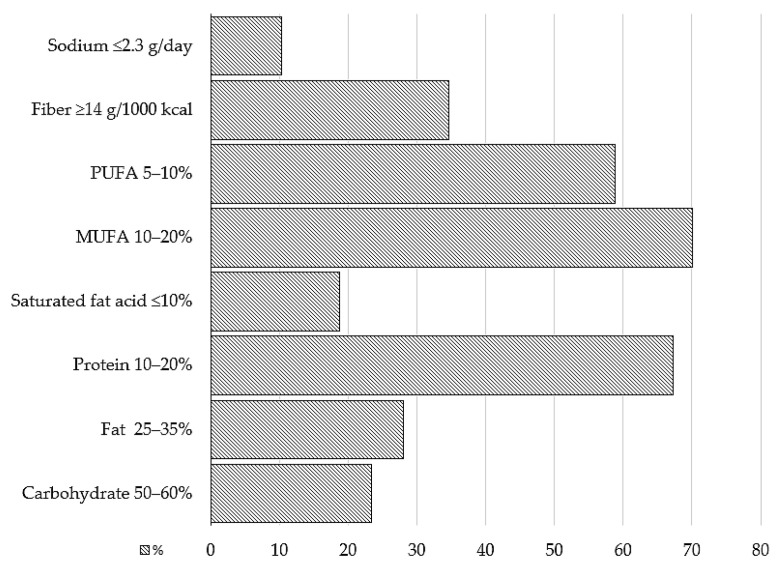
Adherence to specific dietary recommendations. Abbreviations: PUFA, polyunsaturated fatty acids; MUFA, monounsaturated fatty acids; %, the percentage of subjects who adhered to recommendations.

The daily nutrient intake is outlined in Table 2. The majority of participants (33 individuals) simultaneously adhered to three dietary recommendations (Figure 1). Two subjects did not comply with any of the recommendations, and no participant was able to follow all the considered recommendations. Participants most frequently exhibited adherence to the recommended proportions of MUFA and protein, with 75 and 72 individuals, respectively (Figure 2). Conversely, the lowest adherence was observed for the recommended salt intake.

### 3.3. Impact of Adherence to Dietary Recommendations on the Relationship between Intima–Media Thickness and Visfatin

We identified a common dietary pattern characterized by low carbohydrate and fiber but high fat, total fat, saturated fat, and salt intake. Among individuals adhering to this pattern, a statistically significant correlation was observed between IMT and visfatin. Interestingly, the same relationship was also found in subjects whose intake of PUFA and MUFA aligned with the recommended guidelines (Table 3). The PUFA content in the dietary pattern aligning with nutritional recommendations contributed to a statistically significant association between IMT and visfatin concentration.

Consequently, in this context, we delved into the correlation between omega-6 and omega-3 (Ω-6/Ω-3) ratio. The mean ratio of Ω-6/Ω-3 was 6.61 ± 0.63. When the Ω-6/Ω-3 ratio was ≥2, the correlation between IMT and visfatin was statistically significant (*p* = 0.014). However, if the ratio was <2, the correlation was not statistically significant.

In a linear regression analysis, with PUFA intake ≤ 10% or MUFA ≤ 20% of total energy, the visfatin concentration had a statistically significant correlation with carotid artery IMT (*p* = 0.010 and *p* = 0.006, respectively).

### 3.4. Influence of Common Risk Factors and Nonadherence to Dietary Recommendations on the Association between Intima–Media Thickness and Visfatin

Visfatin concentration and carotid artery IMT exhibit a statistically significant correlation (Table 4, Model 1). However, no statistically significant correlation was found between visfatin or IMT and various consumption categories. In the multivariable linear regression analysis, the significance of the associations between visfatin and IMT persisted and even strengthened after adjusting for common risk factors, medication usage, and nonadherence to dietary recommendations (Table 4, Model 3).

## 4. Discussion

This study’s findings indicate that individuals with T2DM exhibit dietary patterns that deviate from general dietary recommendations. A statistically significant association between visfatin and subclinical atherosclerosis is evident alongside these nutritional imbalances. This association persists even after adjusting for common atherosclerotic risk factors and the influence of medication use.

Individuals exhibiting elevated BMI and increased body fat percentage, possessing advanced educational backgrounds, undergoing combined antidiabetic therapies, and demonstrating elevated levels of HbA1c exhibited a greater proclivity for veracious self-reporting of dietary habits. This inclination towards transparent dietary disclosure may indicate a heightened awareness among these subjects regarding the imperative to modify their dietary practices. Notwithstanding this heightened awareness, however, the nutritional composition of the reported food among these individuals did not manifest any substantial disparities when juxtaposed with the comparison group.

This study’s reported food intake composition closely resembled the type 2 diabetes patient’s dietary survey by the Polish Society of Diabetology [8]. However, some discrepancies were noted between studies. This study included higher fiber and fat intake and lower saturated fatty acid and salt intake. In comparison to Japan, our study population exhibited lower carbohydrate but higher fat and protein intake, potentially influencing cardiovascular disease prevalence and life expectancy outcomes [41,42].

While analyzing nutritional data, we applied dietary recommendations from national guidelines and the European Association for Diabetes Research [9,39]. Our findings reveal that none of the subjects adhered to all eight recommendations simultaneously, with the majority following two to three guidelines. Notably, 70% of patients achieved the recommended intake of MUFA, followed by protein and PUFA consumption, while greater deviations were observed with respect to salt, saturated fatty acids, and carbohydrates. This study contrasts with the previously mentioned study, in which adherence to 7 out of 10 recommendations was reported [8]. Our observations may result, at least to some extent, from subjects following outdated diabetes dietary recommendations.

Our previous work established that the proinflammatory cytokine visfatin is a marker for subclinical atherosclerosis in individuals with type 2 diabetes [43]. We identified a significant association between visfatin and the consumption of some macronutrients, indicating the impact of dietary composition on cardiovascular disease in T2DM. To our knowledge, this study is the first demonstration of alterations in the correlation between visfatin and subclinical atherosclerosis when adjusted for adherence or non-adherence to dietary recommendations.

D. de Luis et al. showed that hypocaloric diets reduce visfatin concentration in obese non-diabetic patients [24]. In contrast to us, they did not take into consideration the underlying modified food composition. However, their data hint at a shift towards higher dietary carbohydrate and protein intake and a proportional reduction in fat intake. This aligns with the results obtained in this study.

The association between visfatin concentration and different nutritional strategies has been explored. There is no observed correlation between visfatin levels and Japanese, Westernized, lacto-ovo-vegetarian, or Mediterranean diets [44,45,46]. Nevertheless, adherence to the hypocaloric and Okinawan-based Nordic diets has demonstrated a reduction in visfatin concentration [24,47]. Assessing the impact of visfatin based solely on dietary patterns is challenging, as other factors like alternation in macronutrient composition, salt content, or weight loss may also play a role.

Dietary guidance is vital in T2DM management, impacting macrovascular complications through proinflammatory cytokine activation. Individuals with ≤50% carbohydrate intake in this study showed a positive correlation between visfatin and IMT thickening, aligning with our earlier findings on low carbohydrate intake and subclinical atherosclerosis [35]. This echoes a recent study’s positive association between visfatin and carbohydrate intake, acknowledging limitations like small sample size, younger participants, shorter diabetes duration, and the lack of adjustments for cardiovascular drug usage [23]. Nonetheless, the carbohydrate as a percentage of total energy intake closely mirrored this study’s observations.

A low-carbohydrate diet leads to increased fat intake, impacting plasma membrane function and cellular responses through dietary fatty acids [22,48]. Our findings align with research associating high fat intake with atherosclerosis [22,48], highlighting a positive connection to visfatin in T2DM patients. The fat source may influence the relationship between total dietary fat and subclinical atherosclerosis [6]. This study identified a statistically significant link between visfatin and subclinical atherosclerosis when SFA intake exceeded the recommended level, consistent with observations of increased serum visfatin during an SFA-rich diet [26]. Unfortunately, this study did not permit analysis of specific macronutrient sources. In contrast, studies have demonstrated that SFAs from meat increase CVD risk, while those from vegetables or fish/seafood do not elevate risk [6,12]. Thus, not all fats in the diet can be universally classified as unhealthy.

This study’s findings reveal a positive association between visfatin and carotid artery IMT in individuals whose intake of MUFA and PUFA is at the recommended level. The intriguing aspect is that MUFAs and PUFAs, particularly omega-3 fatty acids, improve lipid profile and endothelial function, lower blood pressure, reduce insulin resistance and inflammation, and exhibit antithrombotic effects [2,11]. Nonetheless, the results of the studies are conflicting. Among overweight individuals, an inverse correlation is described between visfatin and MUFAs, while no association has been identified in those with type 2 diabetes or postmenopausal females [23,25,26,28]. Some studies have found a positive correlation between visfatin and PUFAs without any correlations with omega-3 or omega-6 in patients with T2DM [23]. Some find a positive correlation between omega-6 and visfatin levels [25]. The question may involve the debated omega-6 to omega-3 (Ω-6:Ω-3) ratio. An advised optimal ratio of Ω-6:Ω-3 acids is suggested to range from one to four [49]. In this study, a ratio of ≥2 was linked to a positive association between visfatin and carotid artery IMT. It has been shown that supplementation with 1 g/day of Ω-3 improved glycemic control but had no impact on visfatin levels, while 4 g per day led to a significant 25% reduction in cardiovascular events in T2DM [27,50]. We found that if intake of PUFA ≤ 10% and MUFA ≤ 20% of energy, visfatin was strongly associated with IMT. However, it is vital to emphasize that the limited number of subjects surpassing the recommended intake of PUFAs and MUFAs does not provide sufficient grounds to definitively advocate for the consumption of these fatty acids beyond current recommendations. Still, an insufficient intake of PUFA is considered a crucial deficiency in inadequate diets, and therefore, increasing PUFA intake is more important than reducing the high intake of SFA [15]. The significance of the macronutrient source, whether from animal or plant sources, is often overlooked in existing studies [6,12,14]. Further research with a larger population of patients with T2DM, considering the diverse outcomes and variations in food composition across studies.

In this study, protein intake did not affect the association between visfatin and IMT. While high-protein diets are reported to improve weight loss and HbA1c, their impact on cardiovascular risk factors is inconclusive [51,52]. Notably, there is a gap in the literature regarding the relationship between visfatin and subclinical atherosclerosis concerning protein intake. Dietary fiber, known for its anti-inflammatory properties, is protective against myocardial infarction [53]. Although one study showed no correlation between visfatin and fiber intake [23], our research identified a significant association between visfatin and IMT in individuals with insufficient dietary fiber, supporting existing evidence for the cardiovascular benefits of adequate fiber consumption [54]. Excessive salt intake, linked to hypertension and IMT thickening [55], showed a significant positive correlation with visfatin in this study, highlighting its contributory role as a proinflammatory factor in the complex pathogenesis of IMT thickening.

This study has strengths, including an ethnically homogeneous population, standardized approaches with a single investigator for the radiological studies, deliberate exclusion of under-reporters, and the use of regional food-based software for precise 24 h intake data. The cross-sectional nature of this study limits the establishment of causation. Extrapolating the impact on the extended progression of atherosclerosis from a single day’s dietary record is somewhat provocative, given the modest yet statistically significant correlation. Nevertheless, our data align well with the existing literature. The complex regulation of proinflammatory adipokines under different dietary patterns is still poorly understood and warrants further research, particularly regarding recommended fatty acid limits for type 2 diabetes patients.

## 5. Conclusions

Our dietary choices significantly impact our health, and while a low-carbohydrate diet helps regulate blood sugar levels, it poses risks from increased fat intake and accelerated atherosclerosis in type 2 diabetes. This study identified a pattern with low carbohydrate and fiber but high fat, total fat, saturated fat, and salt intake, intensifying the statistical link between visfatin and subclinical atherosclerosis.

## Figures and Tables

**Table 1:** Clinical characteristics of the study population.

	Total	Under-Reporters	Plausible Reporters	*p*-Value
*n* (%)	212	105 (49.5%)	107 (50.5%)	
Sociodemographic factors				
Men (%)	89 (42.0%)	89 (42.0%)	43 (41.0%)	NS
Age (years)	59.0 ± 8.0	59.0 ± 7.4	58.7 ± 8.6	NS
Education				
Primary	27 (12.7%)	13 (12.4%)	14 (13.1%)	NS
Secondary	61 (28.8%)	34 (32.4%)	27 (25.2%)	NS
Vocational education	69 (32.5%)	38 (36.2%)	31 (29.0%)	NS
Higher	55 (25.9%)	20 (19.0%)	35 (32.7%)	0.023
Lifestyle behaviors				
Never smoker	119 (56.1%)	60 (57.1%)	59 (55.1%)	NS
Previous smoker	52 (24.5%)	24 (22.9%)	28 (26.2%)	NS
Current smoker	41 (19.3%)	21 (20.0%)	20 (18.7%)	NS
Smoking pack years	20.9 ± 14.5	20.2 ± 15.8	21.4 ± 13.4	NS
Physical activity (MET-min/week)	4634.1 ± 270.4	4648.5 ± 359.2	4620.0 ± 405.5	NS
Body composition				
BMI	34.1 ± 5.74	35.6 ± 5.6	32.5 ± 5.5	<0.001
Total Tissue Fat %	39.4 ± 8.1	40.7 ± 7.5	38.2 ± 8.4	0.031
A/G ratio	1.2 ± 0.2	1.2 ± 0.2	1.2 ± 0.2	NS
Past medical history				
Duration of DM (years)	7.1 ± 6.0	6.6 ± 5.6	7.6 ± 6.4	NS
Hypertension	185 (87.3%)	93 (88.6%)	92 (86.0%)	NS
Hyperlipidemia	109 (51.4%)	49 (46.7%)	60 (56.1%)	NS
SBP (mmHg)	146.0 ± 17.8	147.3 ± 16.6	144.7 ± 18.9	NS
DBP (mmHg)	88.6 ± 9.2	89.56 ± 8.7	87.78 ± 9.7	NS
Medication history				
Oral antidiabetic drugs	185 (87.3%)	90 (85.7%)	95 (88.8%)	NS
Metformin	178 (84.0%)	88 (83.8%)	90 (84.1%)	NS
GLP-1 RA	5 (2.4%)	5 (4.8%)	0	0.025
SGLT2I	6 (2.8%)	5 (4.8%)	1 (0.9%)	NS
DPP4I	35 (16.5%)	13 (12.4%)	22 (29.6%)	NS
TZD	2 (0.9%)	1 (1.0%)	1 (0.9%)	NS
SU	62 (29.2%)	24 (22.9%)	38 (35.5%)	0.043
Insulin	20 (9.4%)	7 (6.7%)	13 (12.1%)	NS
Antihypertensive therapy	159 (75.0%)	82 (78.1%)	77 (72.0%)	NS
ACEI or ARB	127 (59.9%)	69 (65.7%)	58 (54.2%)	NS
Beta-blocker	80 (37.7%)	45 (42.9%)	35 (32.7%)	NS
CCB	52 (24.5%)	28 (26.7%)	24 (22.4%)	NS
Antiaggregants *	52 (24.5%)	25 (23.8%)	27 (25.2%)	NS
Lipid-lowering agents	58 (27.4%)	27 (25.7%)	31 (29.0%)	NS
Laboratory				
Total-C (mmol/L)	5.6 ± 1.3	5.7 ± 1.1	5.5 ± 1.4	NS
HDL-C (mmol/L)	1.4 ± 0.3	1.4 ± 0.3	1.4 ± 0.4	NS
LDL-L (mmol/L)	3.5 ± 1.2	3.56 ± 1.1	3.4 ± 1.2	NS
LDL > 2.6 mmol/L	154 (72.6%)	80 (76.2%)	74 (69.2%)	NS
Triglyceride (mmol/L)	2.1 ± 1.21	2.2 ± 1.3	2.1 ± 2.1	NS
Non-HDL-C (mmol/L)	4.2 ±1.2	4.3 ± 1.1	4.1 ± 1.3	NS
Visfatin (pg/mL)	3.05 ± 0.37	3.1 ± 4.81	3.01 ± 0.56	NS
hsCRV (mg/L)	3.9 ± 0.4	3.8 ± 0.5	4.0 ± 0.5	NS
HbA1c (%)	6.9 ± 1.2	6.8 ± 1.2	6.9 ± 1.2	NS
Atherosclerosis markers				
IMT (mm)	0.83 ± 0.13	0.82 ± 0.12	0.84 ± 0.14	NS
Presence of plaque	94 (44.3%)	42 (40,0%)	52 (48.6%)	NS
Mean IMT > 1 mm or plaque	95 (44.8%)	42 (40,0%)	53 (49.5%)	NS
Mean IMT > 1 mm	25 (11.8%)	17 (15.9%)	17 (15.9%)	NS

Data are expressed as mean ± SD or n and %. Abbreviations: NS, non-significant; MET-mins, metabolic equivalent of task minutes; BMI, body mass index; A/G, android-to-gynoid fat ratio; DM, diabetes mellitus; SBP, systolic blood pressure; DBP, diastolic blood pressure; GLP-1 RA, glucagon-like peptide-1 receptor agonists; SGLT2I, sodium–glucose co-transporter-2 inhibitors; DPP4I, dipeptidyl-peptidase-4 inhibitors; TZD, thiazolidinediones; SU, sulphonylureas; ACEI, angiotensin-converting enzyme inhibitors; ARB, angiotensin II receptor blockers; CCB, calcium channel blocker; total-C, total cholesterol; HDL, high-density lipoprotein; LDL, low-density lipoprotein; hsCRP, high-sensitivity C-reactive protein; HbA1c, hemoglobin A1C; IMT, intima–media thickness. * Reported by the participant (prescribed therapy and acetylsalicylic acid).

**Table 2:** Dietary intakes.

	Plausible Reporters
N (%)	107 (50.5%)
Energy intake (kcal)	1857.8 ± 490.1
Carbohydrates (g/d)	212.2 ± 73.1
Carbohydrates (% of total energy)	45.0 ± 11.4
Fats (g/d)	77.6 ± 28.9
Fats (% of total energy)	36.7 ± 9.2
Protein (g/d)	82.8 ± 33.9
Protein (% of total energy)	17.5 ± 5.2
Fiber (g/d)	21.9 ± 9.2
Fiber g/1000 kcal	12.2 ± 5.0
PUFA (g/d)	12.7 ± 6.4
PUFA (% of total energy)	6.0 ± 2.7
MUFA (g/d)	29.4 ± 12.7
MUFA (% of total energy)	13.8 ± 4.3
SFA (g/d)	30.5 ± 12.9
SFA (% of total energy)	14.5 ± 4.8
Trans fatty acid (g/d)	0.3 ± 0.0
Linoleic acid (omega-6) g/d	9.3 ± 0.6
Linolenic acid (omega-3) g/d	2.6 ± 0.4
Salt (g/d)	6.3 ± 3.8

Data are expressed as mean ± SD or n and %. Abbreviations: PUFA, polyunsaturated fatty acid; MUFA, monounsaturated fatty acid; SFA, saturated fatty acid.

**Table 3:** Correlations between intima–media thickness and visfatin.

Selected Cases	N	Visfatin (pg/mL)	IMT (mm)	r	*p*-Value
Carbohydrates ≤ 50%	73	3.25 ± 0.78	0.842 ± 0.138	0.342	0.003
Carbohydrates 50–60%	25	2.34 ± 0.67	0.837 ± 0.143	−0.195	NS
Carbohydrates ≥ 60%	9	2.85 ± 0.71	0.838 ± 0.162	0.310	NS
Fat ≤ 25%	13	2.51 ± 0.56	0.786 ± 0.139	0.561	0.049
Fat 25–35%	30	1.71 ± 0.29	0.865 ± 0.132	0.015	NS
Fat ≥ 35	64	3.71 ± 0.91	0.839 ± 0.142	0.290	0.020
Protein ≤ 10%	3	0.93 ± 0.35	0.713 ± 0.195	0.946	NS
Protein 10–20%	72	2.83 ± 0.46	0.831 ± 0.135	0.221	NS
Protein ≥ 20%	32	3.61 ± 1.57	0.872 ± 0.142	0.295	NS
PUFA ≤ 5%	38	1.96 ± 0.27	0.823 ± 0.138	0.125	NS
PUFA 5–10%	63	3.76 ± 0.93	0.851 ± 0.143	0.292	0.020
PUFA ≥ 10%	6	1.80 ± 0.24	0.837 ± 0.134	0.515	NS
MUFA ≤ 10%	23	2.21 ± 0.38	0.809 ± 0.137	0.292	NS
MUFA 10–20%	75	3.42 ± 0.79	0.846 ± 0.137	0.287	0.012
MUFA ≥ 20%	9	1.61 ± 0.36	0.867 ± 0.178	−0.136	NS
SFA ≤ 10%	20	2.45 ± 0.47	0.825 ± 0.133	0.433	NS
SFA > 10%	87	3.14 ± 0.68	0.844 ± 0.142	0.246	0.021
Sodium ≤ 2.3 mg/d	11	1.35 ± 0.27	0.823 ± 0.120	0.499	NS
Sodium > 2.3 mg/d	96	3.20 ± 0.62	0.842 ± 0.143	0.251	0.013
Fiber ≥ 14/1000 g/kcal	36	1.92 ± 0.31	0.807 ± 0.144	0.089	NS
Fiber < 14/1000 g/kcal	71	3.56 ± 0.83	0.857 ± 0.136	0.285	0.017

Mean ± SD. Bivariant correlation. Pearson correlation coefficient. Abbreviations: N, number of participants; r, Pearson’s r; NS, non-significant; PUFA, polyunsaturated fatty acid; MUFA, monounsaturated fatty acid; SFA, saturated fatty acid.

**Table 4:** Associations between intima–media thickness and visfatin.

	β	*p*-Value
Model 1	0.251	0.009
Model 2	0.235	0.013
Model 3	0.262	0.006

Multivariable linear regression analysis; the dependent variable is the mean IMT. Β, beta coefficient. Model 1 is unadjusted. Model 2 is adjusted for sex, age, duration of diabetes (in years), HbA1c, hypertension (diagnosis of hypertension, blood pressure more than 140/90 mmHg or antihypertensive treatment), hyperlipidemia (diagnosis of hyperlipidemia or low-density lipoprotein cholesterol >2.6 mmol/L or statin therapy), HDL, high-sensitivity C-reactive protein, smoking habits (include former and current smokers), A/G ratio > 1.0, physical activity (MET-mins per week) (R = 0.599; R^2^ = 0.359; F = 4:649; *p* < 0.001). Model 3 is adjusted for the same as Model 2 and carbohydrate consumption < 50% of total energy; fat consumption > 35% of total energy; salt intake > 2.3 g/day; fiber intake < 14 per 1000 kcal/day, saturated fatty acid intake > 10%, number of followed recommendations, metformin, insulin (R = 0.664; R^2^ = 0.441; F = 3.534; *p* > 0.001).

## Data Availability

The data described in the manuscript are available upon reasonable request.
